# The GPCR Connection: Linking Alzheimer's Disease and Glioblastoma

**DOI:** 10.1111/jcmm.71131

**Published:** 2026-04-09

**Authors:** Ana B. Caniceiro, Sofia P. Agostinho, Luiz F. Piochi, Nícia Rosário‐Ferreira, Irina S. Moreira

**Affiliations:** ^1^ CNC‐UC ‐ Center for Neuroscience and Cell Biology University of Coimbra Coimbra Portugal; ^2^ CiBB ‐ Centre for Innovative Biomedicine and Biotechnology University of Coimbra Coimbra Portugal; ^3^ PhD in Biosciences, Department of Life Sciences University of Coimbra Coimbra Portugal; ^4^ Department of Life Sciences University of Coimbra Coimbra Portugal; ^5^ PURR.AI, Rua Pedro Nunes, IPN Incubadora, Ed C Coimbra Portugal; ^6^ Department of Bioengineering, Instituto Superior Técnico Universidade de Lisboa Lisbon Portugal; ^7^ Instituto de Telecomunicações (IT) Lisbon Portugal; ^8^ iBB – Institute for Bioengineering and Biosciences, Instituto Superior Técnico Universidade de Lisboa Lisbon Portugal

**Keywords:** ageing, Alzheimer's disease, cellular senescence, G protein‐coupled receptors, glioblastoma multiforme, Neuroinflammation

## Abstract

Alzheimer's disease (AD) and glioblastoma multiforme (GBM) are biologically distinct age‐related brain disorders with opposing clinical phenotypes. AD is characterised by progressive neurodegeneration and cognitive decline, whereas GBM is characterised by aggressive cellular proliferation and a poor prognosis. Despite these differences, converging evidence indicates that both conditions share molecular pathways and network‐level dysfunction that emerge during brain ageing. Central to this convergence are G protein‐coupled receptors (GPCRs), which act as integrative signalling hubs that regulate inflammation, metabolism, calcium (CA^2+^) homeostasis, and cell survival. In AD, GPCR signalling modulates amyloid‐β production and clearance, Tau phosphorylation, intracellular CA^2+^ dynamics, and glial‐driven neuroinflammation. In contrast, the same receptor families promote tumour growth, angiogenesis, immune evasion, and therapeutic resistance in patients with GBM. Core intracellular cascades, such as PI3K‐AKT–mTOR and MAPK–ERK, are dysregulated in both diseases and function as shared signalling backbones, with outcomes dictated by cellular context rather than receptor identity. CXCR4, LPA₁, and FPR1 exemplify this duality, driving either oncogenic proliferation or neuronal dysfunction, depending on the biological environment. Recent advances in integrative multiomics, computational modelling, artificial intelligence, and organoid systems have revealed GPCR‐centred regulatory nodes and accelerated the identification of druggable targets. Collectively, these findings suggest that AD and GBM, although pathologically antithetical, share a molecular fingerprint shaped by ageing‐associated inflammation, metabolic disruption, cellular senescence and dysregulated GPCR networks. Deciphering this context‐dependent duality may enable precision therapeutic strategies to either restore neuronal integrity in AD or suppress malignant programmes in GBM while fostering cross‐fertilisation between neurodegeneration and neuro‐oncology research.

## 1 Alzheimer's Disease and Glioblastoma Multiforme

Neurodegenerative diseases affect the central nervous system (CNS) throughout life and represent a major health burden, particularly in the ageing population. They lead to progressive disabilities and premature mortality [1]. Among them, Alzheimer's disease (AD) is the most common and leading cause of dementia [2], characterised by progressive cognitive decline accompanied by extracellular amyloid‐beta (Aβ) plaque accumulation and intracellular neurofibrillary Tau tangles (NFT) of hyperphosphorylated Tau (p‐Tau) [2]. Although these hallmarks define AD pathology, recent clinical trials targeting Aβ and Tau have shown limited benefits, exposing the multifactorial nature of the disease and the need to examine additional pathogenic pathways linked to brain ageing [3]. In contrast, glioblastoma multiforme (GBM) is an aggressive grade IV brain tumour with a median survival of 8–15 months despite maximal therapy [4]. It mainly affects older adults and is classified by isocitrate dehydrogenase (IDH) status, with IDH‐wild‐type tumours representing most cases and having the poorest prognoses [5, 6]. GBM exhibits extreme cellular heterogeneity, rapid proliferation, metabolic rewiring, angiogenesis and immune evasion. Standard care includes resection, radiotherapy and temozolomide (TMZ) treatment. However, challenges such as TMZ resistance, a dynamic tumour microenvironment and almost universal relapse persist [5]. Tumour‐ and therapy‐induced genetic alterations further drive GBM evolution and complicate its management [7]. Identifying AD and GBM shared pathways, particularly those involving neuroinflammation, metabolism and cell survival, may reveal novel therapeutic strategies.

Despite the opposing outcomes of neuronal loss in AD and uncontrolled proliferation in GBM, these traditionally considered unrelated diseases share similar molecular features [8]. Epidemiological studies have reported an inverse association between neurodegeneration and cancer incidence, with patients with AD showing a lower cancer risk and individuals with gliomas, especially GBM, a reduced AD risk [8]. Genetic studies have similarly linked glioma‐associated variants to decreased susceptibility to AD. However, the underlying mechanisms remain unclear, and isolated clinical reports have revealed that both conditions can co‐occur [9, 10], suggesting that additional biological and clinical factors may influence this relationship.

Transcriptomic analyses revealed the joint dysregulation of key pathways. While MAPK/ERK signalling is upregulated in GBM and downregulated in AD, angiopoietin signalling displays the opposite pattern, potentially explaining the inverse epidemiological trend [11]. The ERK‐AKT‐p21‐cell cycle and anti‐angiogenic pathways have been proposed as regulatory nodes bridging neurodegeneration and tumorigenesis. However, contrasting studies have reported positive correlations between AD and malignant brain tumour mortality in older adults, suggesting that shared genetic and environmental factors may influence both disorders [12].

Disruptions in cell survival pathways may represent a central molecular link between these two conditions. The PI3K/AKT/mTOR axis regulates neuronal survival, autophagy, and cell cycle re‐entry, and its dysregulation contributes to aberrant neuronal activation in AD and proliferative signalling in GBM [13]. This challenges the amyloid‐centric view of AD and positions PI3K/AKT/mTOR as a potential dual therapeutic target for AD and GBM treatment.

Although neurones are the primary functional units of the CNS, microglia and infiltrating macrophages strongly influence neurodegeneration and tumour progression. These myeloid cells regulate immune activation, metabolic adaptation, blood–brain barrier (BBB) integrity and interact with other CNS cell types. Modulating microglial pathways to enhance immune activation in GBM while reducing neuroinflammation in AD has been proposed as a potential shared therapeutic strategy [14]. NF‐κB signalling, microRNA networks and TREM2‐mediated pathways are important regulators of both conditions [15]. Single‐cell transcriptomics further identified overlapping dysregulated genes related to apoptosis, cell cycle, PI3K/AKT and RAS/MAPK signalling, alongside candidate drugs such as Tubastatin A and vorinostat [16]. RNA‐seq analyses of non‐tumour brain regions in patients with GBM have also revealed accelerated ageing signatures and AD‐like gene expression patterns, emphasising the widespread effects of GBM treatment on the CNS [17, 18].

Taken together, these observations suggest that Alzheimer's disease and glioblastoma multiforme, despite their opposing clinical phenotypes, converge on a shared set of ageing‐modulated signalling backbones. In this context, G protein‐coupled receptors (GPCRs) emerge not as disease‐specific actors but as upstream integrators whose outputs are determined by the cellular state, microenvironment and disease stage [19, 20]. The following sections examine the rationale and therapeutic potential of targeting GPCRs as shared modulators of AD and GBM.

## 2 GPCRs In Neurological Disorders

GPCRs are central regulators of CNS function throughout the lifespan, controlling neurotransmission, synaptic plasticity, neuroinflammation, metabolism, and cell survival [21]. A large fraction of non‐sensory GPCRs are expressed in the brain, where they regulate neuronal excitability and glial responses to extracellular signals [22]. Despite their structural diversity, GPCRs share a conserved seven‐transmembrane architecture and act as flexible signalling nodes that integrate environmental, metabolic, and inflammatory cues [23].

**FIGURE 1:**
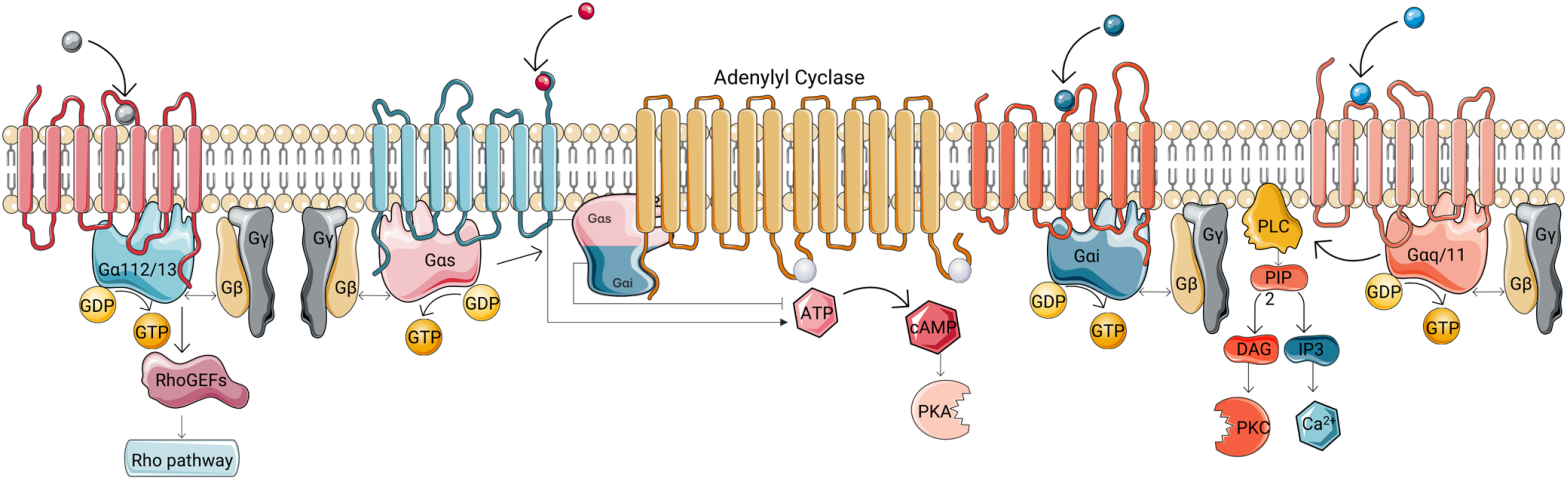
G protein alpha (Gα) subunit signalling pathway. GPCR activation promotes GDP‐GTP exchange in the Gα subunit, initiating family specific responses. Gα12/13 family activates RhoGEFs and Rho pathway signalling, regulating cytoskeletal dynamics and cell migration. Gαs stimulates adenylyl cyclase (AC), increasing cAMP levels and activating the protein kinase A (PKA). Gαi inhibits AC, resulting in reduced cAMP levels and suppression of downstream signalling. Gαq/11 activates phospholipase C (PLC), which hydrolyses phosphatidylinositol 4,5‐bisphosphate (PIP2) into the secondary messenger diacylglycerol (DAG) and inositol triphosphate (IP3). DAG activates protein kinase C (PKC), whereas IP3 increases intracellular calcium (Ca^2+^) levels. Made with Servier Medical Art (CC BY 4.0). This figure was created using Servier Medical Art under a CC BY 4.0 licence.

Upon ligand binding, GPCRs undergo conformational changes that activate heterotrimeric G proteins (Gs, Gi, Gq/11 and G12/13), triggering intracellular cascades that regulate second messengers (Figure 1) such as cAMP, CA^2+^ and Rho‐family GTPases [24, 25]. Beyond canonical G protein signalling, GPCR activity is dynamically shaped by β‐arrestins and G protein‐coupled receptor kinases (GRKs), which control receptor desensitisation, internalisation and non‐canonical signalling [26]. GPCR‐mediated pathways do not typically operate alone but interact extensively with receptor tyrosine kinases [27], ion channels [28], and intracellular kinase cascades, such as MAPK and PI3K–AKT signalling [29]. Additional layers of regulation arise from receptor homo‐ and hetero‐dimerisation [30], basal (ligand‐independent) activity [31], and context‐dependent coupling to downstream effectors. These mechanisms generate interconnected signalling networks in which GPCR activity integrates with other receptor systems to regulate cellular responses. This signalling plasticity enables GPCRs to fine‐tune neuronal and glial functions under physiological conditions but also renders them vulnerable to dysregulation during ageing and disease. Altered GPCR signalling contributes to a broad spectrum of CNS disorders, ranging from neuropsychiatric and neurodegenerative diseases [32] to brain malignancies [33]. Notably, the same GPCRs and downstream pathways can support neuronal survival and circuit stability in one context while promoting proliferation, invasion, and immune evasion in another. This duality positions GPCRs as particularly attractive [34], but mechanistically complex, therapeutic targets in age‐related brain disorders, such as AD and GBM.

## 3 GPCR Signalling in Alzheimer's Disease

AD pathogenesis arises from the interplay of multiple signalling systems, including non‐receptor pathways governing amyloid processing, Tau phosphorylation, mitochondrial dysfunction and cellular stress responses [35, 36, 37]. Within this broader molecular landscape, GPCR signalling acts alongside non‐receptor pathways as an important modulatory layer that integrates extracellular cues and amplifies intracellular signalling cascades associated with disease progression. In this context, GPCR signalling dysregulation has been linked to Aβ accumulation and p‐Tau, the two molecular hallmarks of AD, as well as neuroinflammation, synaptic dysfunction and metabolic dysregulation. GPCRs, which are widely expressed in neurons, astrocytes and microglia, are important regulatory components and potential targets for the development of disease‐modifying therapeutics [38, 39].

A broad range of GPCRs contribute to these processes, including serotonergic (5‐HT_6_) [40], adrenergic (β2‐adrenoceptor and α1‐adrenoceptor) [41], dopaminergic (D_1_R and D_2_R) [42], cannabinoid (CB_1_R and CB_2_R) [43], trace amine‐associated (TAAR) [44, 45], adenosine (A_1_R and A_2A_R) and sphingosine‐1‐phosphate (S1PR) [46, 47, 48]. Collectively, these receptors modulate neuron‐glial communication, second‐messenger cascades, protein aggregation and cytoskeletal organisation. Pharmacological modulation of GPCRs has both functional and cognitive benefits. For example, 5‐HT_6_ antagonists, masupirdine and idalopirdine have improved cognitive outcomes in clinical settings [40], and α1‐adrenoceptor antagonists, such as prazosin, exhibit neuroprotective effects in preclinical studies [41]. Serotonergic dysfunction, adrenergic signalling imbalance and cannabinoid receptor alterations have been associated with synaptic impairment, neuroinflammation and abnormal protein aggregation in AD, whereas metabolic and inflammatory GPCRs, such as S1PR and adenosine receptors, modulate immune responses and neuronal survival.

Although many AD‐associated GPCRs belong to families A and C, evidence suggests that their contribution to disease pathogenesis does not strictly depend on receptor family classification [39, 49]. Additionally, the role of specific GPCRs may vary depending on the disease stage, as some receptors contribute to early pathogenesis, whereas others are more prominent in late‐stage neurodegeneration [50]. Among the family A GPCRs, muscarinic acetylcholine receptors (mAChRs), adenosine receptors, chemokine receptors and adrenergic receptors have been extensively investigated in AD [51, 52, 53]. Similarly, family C GPCRs, particularly GABA receptors and metabotropic glutamate receptors (mGluRs), have gained attention for their roles in modulating synaptic function, neuroinflammation and neurotransmitter homeostasis [50]. These GPCR pathways interact with other signalling mechanisms that regulate amyloid processing, Tau phosphorylation and cellular stress responses, positioning GPCRs among several modulatory nodes within pathogenic networks. Despite these growing insights, further studies are needed to elucidate the precise roles of GPCRs in AD progression and their potential as therapeutic targets.

### 3.1 GPCRs In Aβ and p‐Tau Pathology

**FIGURE 2:**
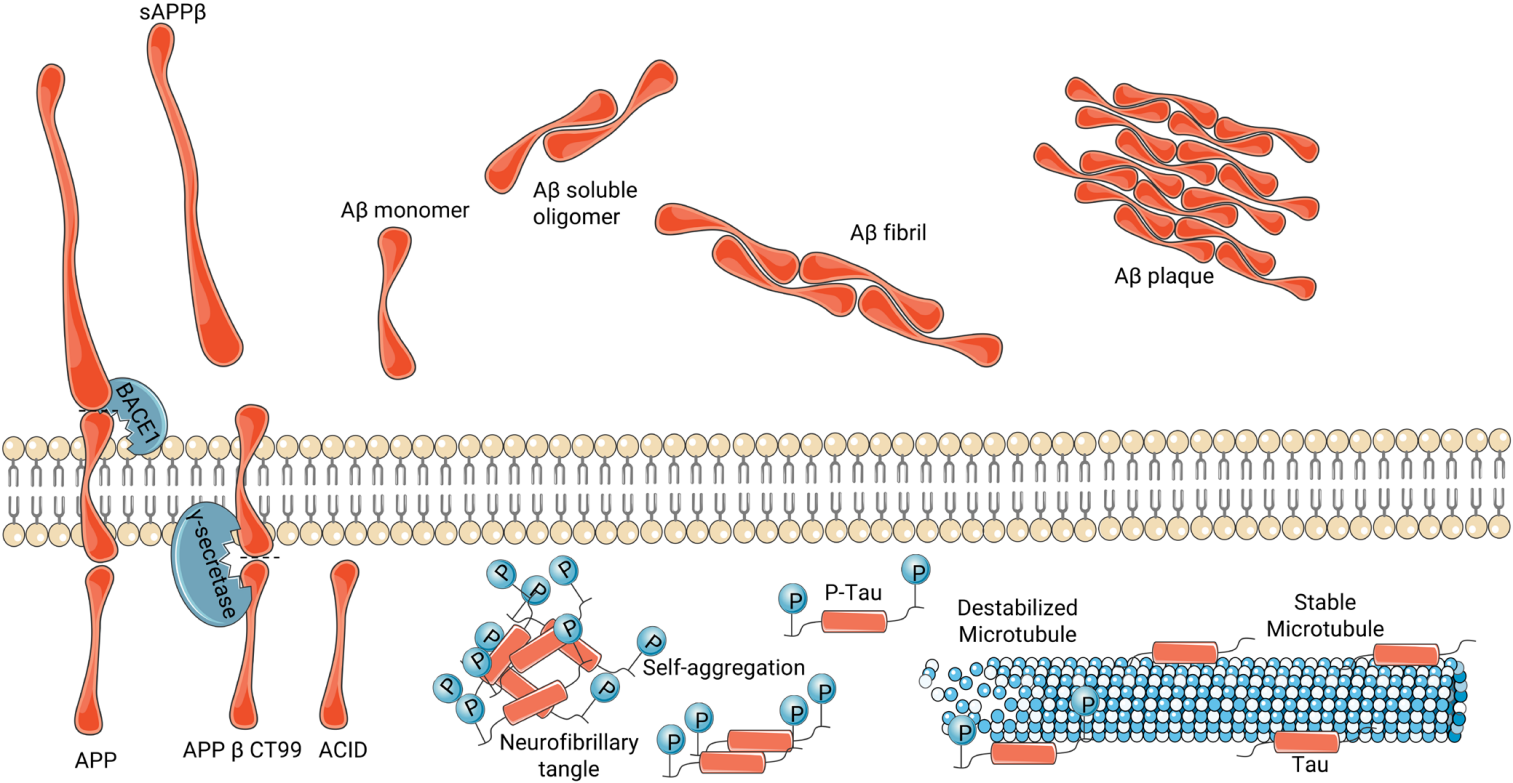
Aβ plaques and NFT formation in patients with AD. Transmembrane APP is first cleaved by BACE1, generating a membrane‐bound APP β C‐terminal fragment 99 (APP β CTF99) and soluble APPβ (sAPPβ). APP β CTF99 is then cleaved by γ‐secretase, producing amyloid precursor protein intracellular domain (AICD) and Aβ monomers, which are secreted outside the cells. Aβ monomers can assemble into soluble oligomers or insoluble fibrils, the latter of which are responsible for Aβ plaque formation in AD. Tau protein naturally attaches to microtubules and stabilizss them. When Tau becomes p‐Tau, it no longer binds to microtubules, rendering them unstable. p‐Tau can self‐aggregate to generate intracellular NFT. Made with Servier Medical Art (CC BY 4.0).

GPCR‐dependent pathways strongly influence inflammatory responses, Aβ plaque production and clearance and NFT formation via p‐Tau (Figure 2). Despite extensive research on AD [54], effective disease‐modifying treatments are limited, and GPCRs are emerging as prime therapeutic targets based on their central role in AD progression.

Aβ is generated through the amyloidogenic processing of amyloid precursor protein (APP), a transmembrane protein that is cleaved sequentially by β‐secretase 1 (BACE1) and γ‐secretase, releasing Aβ monomers that oligomerise into soluble neurotoxic species or form insoluble fibrils that aggregate into amyloid plaques and impair neuronal function [55, 56]. Several GPCRs interact with BACE1 and γ‐secretase, influencing APP processing at multiple sites [39, 57]. The regulatory proteins β‐arrestin 1 and 2 bind to the APH1 subunit of γ‐secretase, enhancing its activity and increasing Aβ production [58]. GRKs 2, 5 and 6, which modulate β‐arrestin interactions, are downregulated in AD, exacerbating amyloidogenesis [59, 60]. The cannabinoid CB_1_ receptor (CB_1_R), a key modulator of synaptic transmission, is downregulated in AD, increasing glutamate release, synaptic hyperexcitability and oxidative stress, leading to APP processing via the amyloidogenic pathway, with consequent upregulation of BACE1 activity and increased Aβ production. CB_2_R activation enhances microglial clearance of Aβ plaques, reducing neuroinflammation and plaque burden, whereas CB_1_R exerts neuroprotective effects by modulating excitotoxicity and oxidative stress [61]. S1P_1_R signalling contributes to neurovascular dysfunction, increasing BBB permeability, immune infiltration and impaired Aβ clearance, thereby worsening AD pathology. Overactivation of S1P_1_R promotes chronic neuroinflammation and endothelial dysfunction, whereas pharmacological modulation using fingolimod shows protective effects in preclinical models by reducing Aβ accumulation and inflammation. Orphan GPCRs, notably GPR3, regulate amyloid processing via γ‐secretase activity and represent promising targets for AD progression modification; their inhibition reduces amyloid pathology in models [62].

Beyond Aβ, GPCRs are deeply involved in Tau pathology, characterised by p‐Tau and aggregation into NFTs that disrupt cytoskeletal integrity and intracellular transport. Tau is phosphorylated by multiple kinase pathways, including those of glycogen synthase kinase‐3 beta (GSK‐3β), cyclin‐dependent kinase 5 (CDK5) and MAPK/ERK pathways [63, 64]. Protective GPCRs, such as muscarinic acetylcholine receptor M1 (M_1_ AChR) and β_2_‐adrenoceptor, reduce p‐Tau levels, whereas stress‐activated GPCRs, such as chemokine (CXCR4) and angiotensin II receptors, promote Tau aggregation [63]. Emerging evidence implicates lysophosphatidic acid receptor 1 (LPA1) as a key driver of Tau pathology; its disruption accelerates p‐Tau, destabilises the cytoskeleton and promotes synaptic degeneration, linking LPA1 dysfunction to neurodegeneration and impaired synaptic remodelling in AD [65, 66].

There is growing evidence that Aβ accumulation induces p‐Tau accumulation, establishing a pathogenic link between these two processes [67]. Transgenic AD mouse studies have shown that Aβ oligomers activate GPCR‐mediated kinase cascades, GSK‐3β and CDK5, accelerating p‐Tau and NFT formation [68]. This feedback loop exacerbates neuroinflammation and synaptic dysfunction, illustrating how GPCRs signalling integrates interconnected AD pathogenic pathways. Targeting specific GPCRs may provide a unique therapeutic avenue for modulating the Aβ‐p‐Tau interconnection.

### 3.2 Calcium Dysregulation and Mitochondrial Metabolism

Calcium (Ca^2+^) is a critical intracellular messenger involved in a wide range of cellular processes, including gene expression, neurotransmission, synaptic plasticity, mitochondrial metabolism and neuronal survival [69]. To maintain cellular homeostasis, Ca^2+^ concentrations are tightly regulated, with cytoplasmic levels being significantly lower than those in the extracellular space and organelles, such as the endoplasmic reticulum (ER) and mitochondria. Controlled intracellular Ca^2+^ release from the ER occurs through inositol 1,4,5‐trisphosphate (IP3) and ryanodine receptors (RyRs), whereas plasma membrane ion channels regulate extracellular Ca^2+^ influx. In AD, dysregulated Ca^2+^ homeostasis disrupts synaptic activity, promotes excitotoxicity and triggers neuronal apoptosis, contributing to the progression of the disease [70]. GPCRs play a pivotal role in Ca^2+^ homeostasis, particularly through the PLC‐IP3‐DAG signalling cascade. Activation of M_1_ AChRs, β_2_‐adrenoceptors and glutamate receptors (mGluRs, NMDA and AMPA) enhances PLC activity, leading to IP3 generation and subsequent Ca^2+^ release from the ER [71, 72]. In AD, abnormal activation of these receptors results in excessive Ca^2+^ influx, which amplifies synaptic dysfunction and promotes neuronal loss. Dysfunctional Ca^2+^ signalling is particularly pronounced in the case of mGluR_5_, a metabotropic mGluR whose aberrant activation is linked to Aβ‐induced excitotoxicity [73, 74]. In transgenic AD models, mGluR_5_ overstimulation leads to sustained Ca^2+^ elevation, exacerbating neuronal stress, impairing synaptic function and causing cell death. Similarly, excessive NMDA receptor activity in AD increases intracellular Ca^2+^ concentrations, further driving excitotoxicity and oxidative stress [49]. These findings reinforce the central role of GPCR‐driven Ca^2+^ imbalances in AD, extending beyond Aβ and p‐Tau formation to include synaptic toxicity and neurodegeneration in the pathogenesis of AD. In addition to its direct influence on synaptic Ca^2+^ signalling, GPCR dysfunction in AD also affects mitochondrial Ca^2+^ homeostasis and energy metabolism, further exacerbating neuronal decline. Mitochondria are key regulators of neuronal ATP synthesis and Ca^2+^ buffering, ensuring proper synaptic transmission and cellular resilience. However, accumulating evidence suggests that GPCR‐mediated Ca^2+^ dysregulation leads to mitochondrial stress and bioenergetic failure, which are hallmarks of AD [75].

One of the major consequences of excessive mitochondrial Ca^2+^ influx is the activation of the mitochondrial permeability transition (MPT), which disrupts the integrity of the mitochondrial membrane. MPT occurs when large permeability transition pores (mPTPs) open in the mitochondrial membrane, allowing the unregulated movement of ions and solutes, leading to membrane depolarisation, ATP depletion and neuronal apoptosis [76]. In AD, heightened Ca^2+^ influx combined with oxidative stress accelerates mPTP opening, initiating a cascade of mitochondrial dysfunction, energy failure and neuronal loss. GPCRs regulate mitochondrial Ca^2+^ uptake, and when GPCR‐mediated Ca^2+^ influx becomes excessive in AD, mitochondrial homeostasis is disturbed, triggering ROS production, oxidative stress and ATP depletion. This energy deficit impairs neuronal function and promotes apoptotic cascades, accelerating neurodegeneration. While controlled mitochondrial Ca^2+^ uptake is essential for oxidative phosphorylation, excessive levels lead to ATP depletion, mitochondrial outer membrane permeabilization (MOMP) and cytochrome c release, ultimately activating caspase‐dependent apoptosis [75].

Sustained GPCR‐driven Ca^2+^ dysregulation compromises mitochondrial homeostasis by promoting excessive Ca^2+^ uptake, reactive oxygen species generation, and ATP depletion. This bioenergetic failure amplifies oxidative stress, disrupts synaptic function and increases susceptibility to apoptotic cell death, establishing a self‐perpetuating cycle of neuronal vulnerability characteristic of AD [76].

### 3.3 Glial GPCRs in AD

Glial GPCR signalling is critical in AD, with astrocytes and microglia influencing disease progression through their interactions with neurones. Astrocytes, once seen mainly as structural support cells, are now recognised as key regulators of synaptic transmission, neurotransmitter recycling and inflammation. In AD, astrocytes become reactive, increasing inflammatory signalling, altering neurotransmitter buffering, and dysregulating GPCR expression [77]. In mouse models of AD, astrocytes upregulate inflammatory response genes while downregulating neurogenesis and neuronal support pathways [78]. Dysregulation of glutamate and GABA transport exacerbates excitotoxicity and synaptic dysfunction, as astrocytes abnormally release glutamate and convert GABA to glutamine, activating mGluR and GABA‐B GPCRs and promoting Aβ and p‐Tau production [79].

Notably, mGluR_5_ expression is significantly increased in astrocytes surrounding Aβ plaques, where Aβ oligomers bind to mGluR_5_ and disrupt normal signalling cascades, triggering pro‐inflammatory responses and amplifying neurotoxicity [80]. Elevated intracellular Ca^2+^ levels in astrocytes mirror neuronal dysregulation in AD [81], likely involving aberrant GPCR signalling that contributes to excitotoxicity in AD. Astrocytes also regulate neurovascular coupling via mGluR_5_‐and Ca^2+^‐mediated pathways that modulate cerebral blood flow. Dysfunctional GPCR signalling in astrocytes may underlie AD‐associated vascular and metabolic disturbances [82]. Despite their neurotoxic effects, astrocytes are neuroprotective. Activation of mGluR_3_, which is reduced in AD, promotes non‐amyloidogenic APP processing, inhibits BACE1 activity, and enhances soluble APPα production, facilitating Aβ_42_ oligomer clearance [80]. This dual nature of astrocytic involvement suggests that the selective modulation of GPCRs may be a therapeutic strategy for mitigating neurotoxicity while preserving the neuroprotective functions.

Microglia, the primary immune cells of the CNS, remove cellular debris, misfolded proteins, and apoptotic neurones through phagocytosis. They become chronically activated and pro‐inflammatory in AD, aggravating the neuronal damage. Microglia are often broadly categorised into two functionally distinct subtypes: M1 microglia, which release pro‐inflammatory cytokines and contribute to neurotoxicity, and M2 microglia, which secrete anti‐inflammatory factors and neurotrophic molecules [83]. Evidence shows that Aβ and Tau aggregates directly activate microglial GPCRs, promoting inflammation and accelerating neurodegeneration. The upregulation of chemokine receptors, mGluRs, and trace amine‐associated receptor 1 (TA_1_R) in microglia has been linked to elevated cytokine production, synaptic dysfunction, and amyloid‐beta (Aβ) accumulation [39, 49, 83]. Increased mGluR_1_ expression near Aβ plaques is associated with synaptic impairment and cognitive deficits in AD models [80].

The inflammatory microglial phenotype in AD also drives reactive astrocytosis, creating a feed‐forward loop that enhances Aβ production through increased BACE1 and γ‐secretase activities [77]. This crosstalk among microglia, astrocytes, and neurones suggests that AD‐associated neuroinflammation is not cell‐autonomous but a coordinated dysfunction across glial populations. Although microglia are often pro‐inflammatory in AD, several microglial GPCRs exert neuroprotective effects. mGluR_5_ activation reduces oxidative stress and dampens the release of inflammatory mediators, promoting an M2‐like phenotype [80]. Similarly, in vitro activation of TA1R by 3‐iodothyronamine induces anti‐inflammatory signalling that counteracts M1 activation and mitigates Aβ‐induced neurotoxicity [84]. These findings support the use of microglial GPCRs as promising therapeutic targets for modulating neuroinflammation while preserving essential immune functions.

The involvement of astrocytes and microglia in AD highlights the importance of GPCR signalling in addition to neurones. GPCR dysregulation in astrocytes drives neurotransmitter imbalance, CA^2+^ disturbances, and neurovascular dysfunction, whereas GPCR‐dependent microglial activation drives chronic neuroinflammation and worsens Aβ and Tau pathology. However, some receptors, including mGluR_3_ in astrocytes and mGluR_5_ and TA_1_R in microglia, exert neuroprotective roles, underscoring the value of selective GPCR‐targeted therapies. Therefore, modulating glial GPCR activity may attenuate neuroinflammation and synaptic impairment, offering a promising disease‐modifying strategy for AD treatment.

While GPCR dysregulation in AD culminates in synaptic failure and progressive neuronal loss, the same signalling architecture is repurposed in GBM to sustain cell survival, proliferation, and invasion. This divergence illustrates the context‐dependent nature of GPCR‐mediated pathways, which can either destabilise post‐mitotic neural circuits or promote malignant growth. Understanding how GPCR networks are redeployed in GBM is vital to fully understand their dual roles in ageing‐related brain pathologies.

## 4 GPCR Signalling in Glioblastoma

GBM is the most aggressive and lethal primary brain tumour, with a median survival of approximately 15 months and a 5‐year survival rate of only 9% despite aggressive treatment strategies [85]. GBM exhibits extensive intratumoral heterogeneity driven by multiple genetic and epigenetic alterations, resulting in highly adaptive oncogenic pathways that promote uncontrolled proliferation, immune evasion, metabolic reprogramming and resistance to therapy [86]. These processes emerge from complex signalling networks involving GPCR pathways and major non‐GPCR mechanisms, including EGFR amplification or mutation, PTEN loss, TP53 alterations and IDH‐associated metabolic rewiring [87]. Within this oncogenic landscape, GPCRs function as one of several signalling hubs, acting in a context‐dependent manner to modulate, amplify, and integrate extracellular and microenvironmental signals associated with tumour progression. Furthermore, GPCRs are emerging as key regulators of GBM pathophysiology, with multiple GPCR‐mediated signalling cascades implicated in tumour growth, angiogenesis, migration, invasion and immune suppression [88, 89], making them potential molecular targets for understanding and disrupting GBM progression [90].

### 4.1 GPCR‐Mediated Proliferation and Survival in GBM

GBM cells exploit GPCR signalling to sustain their proliferation and evade cell death. GPCRs, such as D_2_R, neurokinin‐1 receptor (NK1R) and thrombin receptors, have been shown to drive GBM cell growth by activating pro‐survival signalling pathways. D_2_R is overexpressed in GBM cells, stimulating the GNAI2/Rap1/Ras/ERK axis and promoting cell proliferation and tumour growth [91]. Consequently, selective antagonism of D_2_R (e.g., ONC201) blocks AKT/ERK signalling and induces apoptosis and is currently under investigation in clinical trials for GBM [92]. Similarly, NK1R is upregulated in GBM and facilitates mitogenic signalling via ERK1/2 phosphorylation, enhancing tumour growth and migration [93]. In contrast, serotonin receptors (5‐HT_2A_ and 5‐HT_6_), which are involved in synaptic plasticity and mood regulation, have been shown to enhance GBM cell migration and invasive behaviour, further exacerbating tumour invasiveness [94]. Adrenergic receptors contribute to GBM progression by modulating stress response pathways. Chronic adrenergic stress signalling promotes tumour cell survival and enhances the glioma microenvironment by regulating angiogenic factors, such as vascular endothelial growth factor (VEGF), contributing to therapy resistance. The β_2_‐adrenoceptor, which has been linked to increased Aβ pathology in AD, also facilitates tumour growth in GBM, highlighting its dual role in CNS pathologies [41].

In addition to neurotransmitter GPCRs, thrombin receptors play a crucial role in GBM progression by binding to their receptor which induces RhoA and phospholipase D (PLD)‐mediated activation of Rap1A, promoting cell adhesion, migration and invasion via β1 integrin signalling [95]. The metabotropic P2Y_12_ receptor (P2Y_12_R), which is overexpressed in GBM cells, has also been shown to increase proliferation in the presence of the agonist adenosine diphosphate (ADP), which activates ERK 1/2 through the RhoA/PKC/Raf1/MEK cascade [96]. GPCR‐mediated activation of myocardin‐related transcription factor A (MRTF‐A) and YAP/TEAD transcriptional programmes further amplifies tumour proliferation and invasion [97]. Additionally, GBM cells exhibit altered glucose metabolism, which enhances tumour aggressiveness. High‐glucose conditions have been shown to upregulate formyl peptide receptor 1 (FPR1) and epidermal growth factor receptor (EGFR), increasing cell proliferation, VEGF production and resistance to apoptosis [98].

### 4.2 GPCRs In Angiogenesis

Unrestricted angiogenesis, a hallmark of GBM, is critical for sustaining tumour growth in the hypoxic brain microenvironment [99]. Several GPCRs modulate VEGF‐driven angiogenesis, enhancing vascular permeability, endothelial proliferation and vessel formation [100]. Among these, the endothelial differentiation gene (EDG) family of lysophospholipid receptors (S1P_1‐5_R) is strongly implicated in angiogenesis and tumour progression. S1P receptors signal via the ERK, JNK, Rho/Rac and PI3K pathways, facilitating endothelial migration and vascular remodelling [90]. Additionally, the chemokine receptors CXCR4 and CXCR5 have been implicated in VEGF‐dependent angiogenesis, whereas orphan GPCRs GPR126 and GPR124 have been identified as novel regulators of vascular invasion in GBM [101]. A particularly relevant overexpressed GPCR in GBM angiogenesis is epidermal growth factor, latrophilin, and seven‐transmembrane domain‐containing protein 1 (ELTD1), which is associated with poor prognosis. ELTD1 interacts functionally with VEGFR2 to enhance vascular proliferation and tumour progression [102, 103]. High levels of EGFR have also been identified in GBM, along with the overexpression of TF, PAR1 and PAR2, and ectopic synthesis of factor VII (FVII), which in turn causes the overproduction of angiogenic factors such as VEGF and IL‐8 [104].

### 4.3 Invasion and Immune Evasion

GBM is highly invasive, with tumour cells infiltrating the surrounding brain regions and evading immune surveillance. Several GPCRs orchestrate cytoskeletal remodelling, extracellular matrix degradation and immune suppression, thereby enabling unrestricted tumour invasion. Chemokine receptors, such as CXCR4 and CXCR7, are central to GBM pathophysiology. CXCR4 is overexpressed in glioma stem cells, maintaining stemness and promoting recurrence, whereas CXCR7 aids in invasion and apoptosis resistance in hypoxic regions [105]. Both are considered therapeutic targets, as they drive directional migration towards CXCL12‐rich hypoxic niches [105], with the CXCR4 inhibitor plerixafor being clinically tested. Formyl‐peptide receptors FPR1‐3 promote chemotaxis, matrix metalloproteinase release, and secretion of pro‐angiogenic factors, accelerating dissemination [85, 106, 107]. Among lysophospholipid receptors, LPA signalling enhances motility, EMT‐like transitions, and therapy resistance, whereas S1P signalling shapes the immunosuppressive tumour microenvironment [108]. GPCR‐mediated immune evasion is further reinforced by cannabinoid receptors CB_1_R/CB_2_R, whose overexpression dampens antitumour immunity but can trigger cell cycle arrest and apoptosis under experimental agonism [109]. Finally, the orphan receptor GPR17 is a potential therapeutic target as a negative regulator of GBM growth, as its overexpression increases reactive oxygen species, induces apoptosis, and suppresses proliferation [108].

### 4.4 Therapeutic Targeting of GPCRs in GBM

Given their widespread dysregulation in GBM, GPCRs are highly druggable targets for therapeutic interventions. Several GPCRs, including CXCR4, FPR1, LPA receptors, S1P receptors, and cannabinoid receptors, have been investigated as potential therapeutic targets for GBM treatment. Targeting CXCR4 and CXCR7 with small‐molecule inhibitors has shown promise in preclinical models, reducing GBM cell invasion and stemness [105]. Similarly, antagonists of LPA and S1P have been explored for their potential to suppress GBM proliferation and angiogenesis [108]. Cannabinoid‐based therapies, such as CB_2_‐selective agonists, exhibit antitumour effects by promoting apoptosis and reducing GBM stem cell viability [109]. Additionally, NK1R antagonists effectively reduce GBM cell migration and invasion [110]. Given the complex nature of GPCR signalling mechanisms and the possibility of side effects when modulating their activity, biased signalling presents an opportunity to enhance the effectiveness of GPCR therapies in GBM. Representative examples include sphingomyelin phosphodiesterase 1 inhibition with fluoxetine to inhibit EGFR and activate lysosomal stress to kill GBM cells [111], and cholesterol depletion using methyl‐β‐cyclodextrin, which inhibits EGF with further impairment of downstream signalling, reducing the sustained activation of both AKT and ERK1/2 in GBM cells [112]. Despite promising preclinical advances, the high heterogeneity and adaptive capacity of GBM remain major obstacles in the development of GPCR‐targeted therapies. However, accumulating evidence indicates that GPCR signalling underpins virtually every hallmark of GBM. Strikingly, many of the same receptors and downstream pathways are implicated in AD, yet they drive diametrically opposed biological outcomes. This paradox raises a fundamental question: how can identical GPCR networks orchestrate both neurodegeneration and malignancy?

## 5 Converging Pathways: GPCRs Linking GBM and AD

**FIGURE 3:**
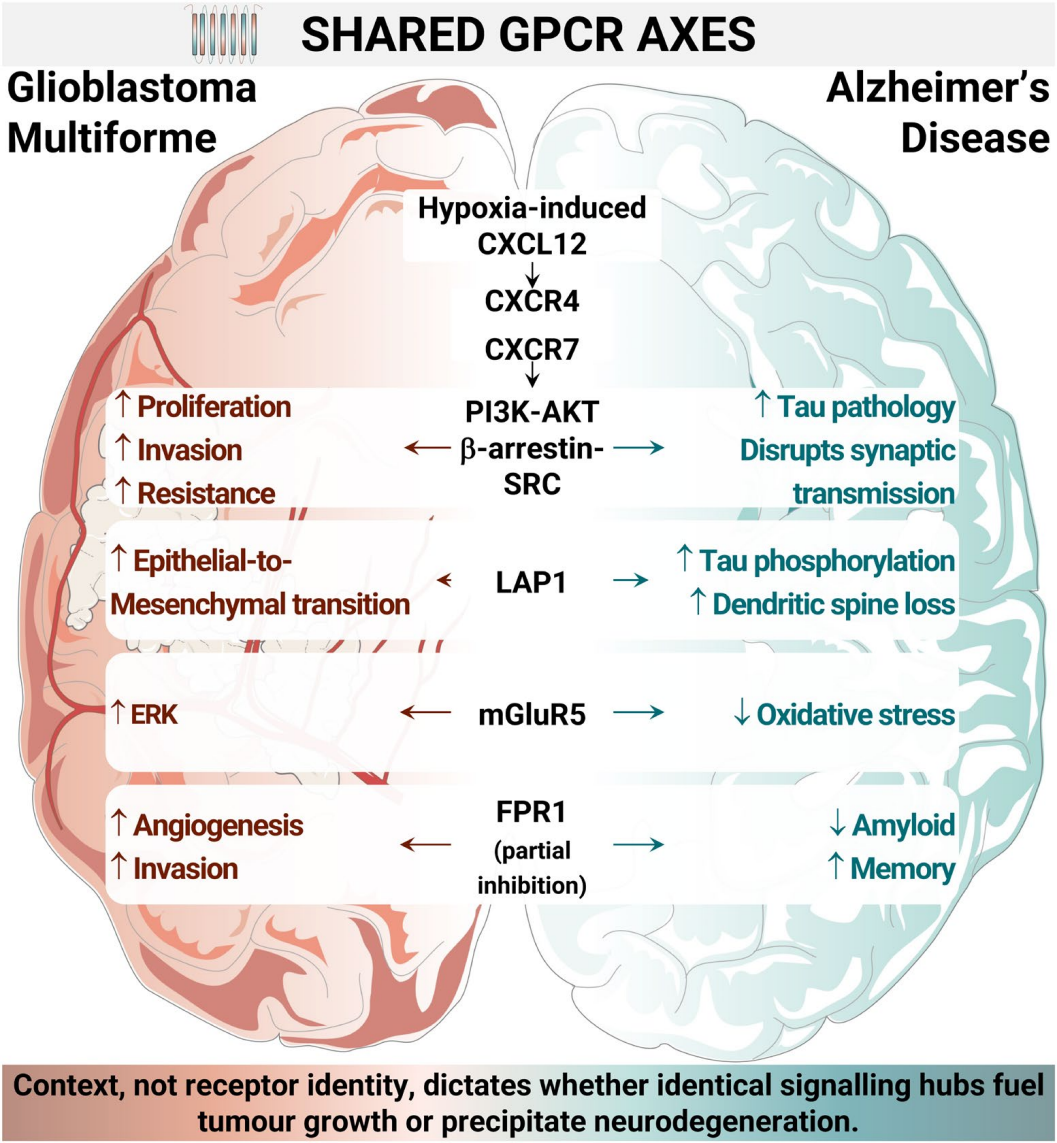
Context‐dependent divergence of shared GPCR signalling in AD and GBM. Common GPCRs (CXCR4, LPA₁, FPR1, mGluR5) converge on intracellular cascades including PI3K‐AKT, MAPK–ERK and β‐arrestin‐SRC. These shared hubs yield disease‐specific outcomes: Proliferation, invasion and EMT in GBM versus synaptic degeneration, Tau pathology and oxidative damage in AD. The cellular context determines whether a pathway fuels malignancy or leads to cell death. Made with Servier Medical Art (CC BY 4.0).

Epidemiological data and transcriptomics analyses have increasingly linked AD and GBM through inverse comorbidity, in which individuals who develop one disorder show a reduced incidence of the other disorder [113]. However, such epidemiological associations may be influenced by confounding factors, such as survival bias, treatment exposure and diagnostic bias. Despite their opposing clinical trajectories, neurodegeneration and malignancy converge on a common set of GPCRs and downstream signalling hubs, including the PI3K/AKT/mTOR, MAPK/ERK, JAK–STAT, Wnt/β‐catenin and NF‐κB pathways [11, 13, 15]. These cascades govern cell survival, immune activation, and metabolic homeostasis in both neuronal and tumour cell contexts. Figure 3 outlines these shared molecular axes and positions GPCRs as upstream integrators capable of driving divergent outcomes depending on the cellular context.

This overlap becomes mechanistically tangible when individual receptors are examined in detail. In GBM stem‐like cells, CXCR4 (often paired with CXCR7) is activated by hypoxia‐induced CXCL12, triggering PI3K/AKT and β‐arrestin‐SRC scaffolding, which supports proliferation, invasion and resistance to therapy [105]. In AD, the same receptor axis in microglia and neurones contributes to synaptic dysfunction and accelerates tau‐related pathology. Similarly, LPA1 drives EMT in tumours via Gq/11‐mediated Rho activation, whereas in the AD brain, LPA1 overactivation promotes p‐Tau accumulation and dendritic spine degeneration [59, 60]. Likewise, FPR1 promotes angiogenesis and extracellular matrix remodelling through VEGF and MMP upregulation in GBM [106, 107] while in AD, its partial inhibition reduces the Aβ bu. and rescues memory function [52]. Even mGluR5 shows this context‐dependent behaviour, activating mitogenic ERK signalling in glioma cells but offering neuroprotection in AD models by attenuating oxidative stress in astrocytes and in microglia [80, 82]. These examples demonstrate that identical receptor‐pathway architectures can yield contrasting physiological consequences in different contexts. In the tumour microenvironment, sustained activation of PI3K/AKT or ERK via CXCR4 or LPA1 drives cell cycle re‐entry, angiogenesis, and metabolic reprogramming in tumour cells. In contrast, in post‐mitotic neurones, these signals disrupt cytoskeletal integrity, impair mitochondrial function and trigger neurodegeneration [58]. Ultimately, it is the cellular context, not the receptor identity, that determines whether a signalling cascade sustains proliferation or precipitates collapse.

## 6 Discovery Platforms for GPCR Targets in AD and GBM

The search for druggable GPCRs in AD and GBM has shifted from gene‐by‐gene studies to integrated, data‐driven approaches. Advances in omics, network inference, machine learning (ML), docking and molecular dynamics have identified GPCRs as key players in neuroinflammation, metabolism and tumour progression [114]. Expression profiling, ligand‐binding assays, CRISPR screens, single‐cell sequencing and organoid models can be used to validate whether computational hits affect Aβ processing, p‐Tau and GBM cell behaviour [115, 116, 117]. Together, these approaches form an iterative in silico‐in vitro‐in vivo workflow, further refined by next‐generation sequencing platforms. Multiomics strengthens target prioritisation, AI tools assess receptor‐ligand interactions and high‐content phenotyping in brain organoids links targets to functional responses. Integrating predictive modelling with experimental testing broadens the GPCR target space and accelerates the transition from computational candidates to translational leads. Notably, these discovery platforms are gaining traction in ageing‐associated datasets to identify GPCRs whose signalling biases arise specifically under chronic inflammation, metabolic stress and cellular senescence.

### 6.1 Computational Approaches

Conventional GPCR target screens are slow, expensive and limited; thus, current pipelines begin with a computational triage. Network‐based analyses of protein interactions and pathway enrichment maps identified receptors embedded in the disease modules. For example, CXCL12 has been linked to AD and GPR17 to GBM using such approaches [118, 119]. ML models trained on genomic or transcriptomic data prioritise GPCRs based on disease relevance, linking several loci to AD risk [120] and identifying CXCR4 as a key GBM driver [121]. Virtual screening approaches, including pharmacophores, docking and Quantitative Structure–Activity Relationship (QSAR), have matched chemotypes to several AD‐upregulated receptors, such as M_5_ AChR, CysLT_2_R, D_5_R, GAL_1_R and 5‐HT_2C_ [122].

Structure‐based discovery leverages high‐quality homology models. Docking followed by molecular dynamics produced nanomolar GPR17 agonists for GBM models [123], whereas hybrid QSAR‐docking workflows were applied to somatostatin receptor 4 and M1 AChR [124, 125]. Transcriptome‐wide differential expression and GSEA are vital for identifying genes that contribute to GBM progression [121]. Increasingly, multiomics integration is used to train neural network QSAR models, as in the optimisation of 5‐HT_6_ antagonists [126]. Currently, each computational hit is directly fed into CRISPR perturbation, live‐cell biosensors and organoid assays, ensuring that only biologically robust GPCRs progress to subsequent stages of target evaluation and drug discovery.

### 6.2 Experimental Validation

Computational shortlists gain translational value only after candidate receptors pass sequential assays that confirm expression, ligand ability, and functional impact in disease‐relevant tissues. Transcript surveys, now commonly extended to single‐cell and spatial RNA‐seq, determine GPCR upregulation or downregulation in AD and GBM. For instance, Huang et al. identified GPR3 among several receptors that are differentially expressed in AD, with validation at the protein level [62]. Similarly, Ganesh et al. reported elevated GPR56 levels in GBM cell lines, linking its abundance to therapy resistance [127]. Next, receptor properties are characterised through ligand‐binding assays, such as radioligand saturation, fluorescence polarisation and NanoBRET. Using these methods, Kari et al. identified sacubitril as a full GPR17 agonist [123]. Functional readouts, such as CA^2+^ flux, cAMP, β‐arrestin recruitment and real‐time conformational biosensors, probe downstream signalling. Nguyen et al. showed that CHBC agonists drive GPR17‐dependent MAPK activation, leading to GBM cell death [128]. Finally, in vivo genetics and pharmacology close the loop: targeted GPR3 deletion in transgenic mice lowers Aβ load and improves cognition, confirming that earlier in silico and cellular findings translate into organismal benefits [62]. This expression‐to‐mechanism‐to‐phenotype pipeline now defines the standards for the development of GPCRs therapeutic targets.

### 6.3 Current GPCR‐Targeted Therapeutics

Despite the absence of AD or GBM‐approved GPCR‐directed drugs, GPCRs remain central therapeutic targets because they regulate synaptic plasticity, inflammation, metabolism and cell cycle regulation. GPCR signalling pathways represent potential therapeutic intervention points that can guide future drug development strategies. In GBM, the strategy is inhibitory: CXCR4 antagonists, such as plerixafor, reduce invasion [105]; ONC201 blocks D2R, inducing apoptosis [91]; and LPA1 inhibitors disrupt glioma‐microglia crosstalk [108]. Conversely, in AD, several GPCRs are enhanced or biased towards protective signals. mGluR_5_ positive modulators support synaptic resilience [80], whereas FPR1 agonism promotes Aβ clearance [52]. Understanding the differences in GPCR conformation and signalling partners between tumours and neurodegenerative tissues may facilitate target prioritisation and structure‐based optimisation of future ligands designed to exploit this context‐specific duality.

In AD, therapeutic efforts aim to restore neurotransmission. M1/M4 potentiators [51, 129, 130, 131, 132], D_1_R/D_2_R stabilisers [133, 134, 135], 5‐HT_6_/5‐HT_2A/C_ antagonists [40, 136], and A2A inhibitors [46, 48, 137] improve cognition and plasticity. Other interventions target hallmark pathologies by reducing GPR3, LPA/S1P or CXCR2 signalling or dampening β2‐adrenoceptor activity, thereby reducing Aβ production, enhancing clearance, and limiting p‐Tau [62, 63, 138, 139]. As metabolic dysfunction accelerates neuronal loss, agents that reduce mGluR_5_ hyperactivity [80, 82] or normalise adrenergic tone [72] are being evaluated for mitochondrial protection.

In GBM, GPCR inhibition aims to block proliferation, invasion and immune evasion processes. Examples include D_2_R blockade with ONC201, inhibitors of S1P1/3 and LPA1/3 [140, 141], and formyl peptide receptor antagonists that reduce VEGF release [107]. Anti‐angiogenic strategies involve the use of CXCR4 or CXCR7 inhibitors and GPR124‐targeting antibodies to restrict tumour vascular support [101, 142, 143, 144]. Modulating CB_2_R or CXCR4 signalling may also counteract immunosuppression by re‐educating tumour‐associated macrophages and improving lymphocyte access [105, 109, 145]. As glioma stem cells drive recurrence, receptors such as GPR56 and those involved in LPA signalling are emerging as attractive targets [127, 146]. Ongoing preclinical and early phase clinical efforts are summarised in Tables 1 and 2, respectively.

**TABLE 1:** GPCR are potential therapeutic targets for AD and GBM, with respective drug candidates, ligand types, biological roles, and clinical trial IDS.

Therapeutic target	Drug candidate	Type of ligand	Clinical trial phase	Biological roles	ClinicalTrials.gov ID
**Alzheimer's Disease**					
α_1_‐adrenoceptor	Prazosin	Antagonist	Phase II	Prazosin acts on α_1_‐adrenoceptors and has been hypothesised to alleviate agitation symptoms.	NCT03710642
A_2_AR	Caffeine	Antagonist	Phase III	Caffeine reduced tau hyperphosphorylation in the hippocampus, alleviated neuroinflammation, and reversed memory loss associated with these effects.	NCT04570085
mGluR_5_	RVT‐101	Antagonist	Phase III	RVT‐101 is effective as an adjunctive therapy to donepezil for treating patients with AD.	NCT02585934
D_2_R	AVP‐786	Partial Agonist	Phase III	AVP‐786 is used to treat agitation in participants with Alzheimer's dementia.	NCT04464564
5‐HT_2A_	Pimavanserin	Inverse Agonist	Phase II	Pimavanserin demonstrates efficacy in treating psychosis associated with AD, exhibiting an acceptable tolerability profile and without negatively affecting cognition.	NCT02035553
M_1_ AChR	HTL9936	Partial Agonist	Phase I	HTL9936 treats memory loss in AD.	NCT02291783
D_2_R, D_3_R, 5‐HT_1A_	Aripiprazole	Partial Agonist	Phase IV	Aripiprazole is used to treat psychosis associated with Alzheimer's disease.	NCT00041678
5‐HT_6_	Masupirdine	Antagonist	Phase II	Masupirdine has been evaluated in patients with moderate AD.	NCT02580305
5‐HT_6_	Idalopirdine	Antagonist	Phase II	Idalopirdine was administered to patients with moderate AD.	NCT01019421
5‐HT_2A_	ACP‐204	Inverse Agonist	Phase II/III	The precise mechanism of action is unclear, but it is described as a next‐generation 5‐HT_2A_ blocker.	NCT06159673
S1P subtypes 1 and 5	Siponimod	Antagonist	Phase II	Siponimod inhibits lymphocyte migration from lymphoid tissues to the central nervous system. Also, pre‐clinical studies revealed that the drug inhibits demyelination and attenuates the production of TNFα, IL‐6, and IL‐17 by astrocytes and microglia	NCT06639282
mGluR5	BMS‐984923	Silent Allosteric Modulator	Phase I	Blocker of amyloid beta oligomer (Aβo)/prion protein (PrPc) toxicity without affecting normal glutamate signalling	NCT06309147
**Glioblastoma Multiforme**					
D_2_R	ONC201	Antagonist	Phase III	ONC201 inhibits cell growth.	NCT04629209
D_2_R	ONC206	Antagonist	Phase I	ONC206 inhibits cell growth.	NCT04541082
MC_4_R	Radio and chemotherapy		Phase III	The study revealed that patients treated with radiotherapy and TMZ with the MC4R rs489693 genotype homozygous for allele A exhibited significantly shorter progression‐free survival and overall survival.	NCT02458508
Smo receptor	Vismodegib	Antagonist	Phase II	Vismodegib exhibits antitumor activity in GBM patients.	NCT00980343
CXCR4	Plerizaflor	Antagonist	Phase II	Plerizaflor inhibits tumour growth and recurrence.	NCT01977677
CXCR4	PRX177561	Antagonist	Phase I	PRX177561 inhibits tumour growth and increases the efficacy of combination therapy.	NCT02765165
D_2_R	Haloperidol (+TMZ)	Antagonist	Phase II	D_2_R overexpression in recurrent GBM mediates Ferroptosis inhibition and chemoresistance. Haloperidol can attenuate the function of D_2_R and increase the sensitivity of GBM to TMZ.	NCT06218524
CB1 and CB2	TN‐TC11G (+ TMZ and Radiotherapy)	Agonist	Phase I/II	TN‐TC11G (THC + CBD) have shown synergistic antitumor effects with temozolomide and radiotherapy in preclinical glioma models.	NCT03529448
CB1	Nabiximols (+ TMZ)	Negative Allosteric	Phase II	Studies have found that CBD and THC reduce tumour growth in animal models of glioma by inducing cell death, inhibiting cell proliferation, and exerting anti‐angiogenic effects.	NCT05629702

Abbreviations: 5‐HT_1A_, 5‐HT receptor 1A; 5‐HT_2A_, 5‐HT receptor 2A; 5‐HT_6_–5‐HT receptor 6; A_2_AR, adenosine A2A receptor; AD, Alzheimer's Disease; CB1, CANNABINOID RECEPTOR Type 1; CB2, cannabinoid receptor Type 2; CXCR4, chemokine receptor Type 4; D_2_R, dopamine receptor D2; D_3_R, dopamine receptor D3; GBM, glioblastoma multiforme; M_1_ AChR, muscarinic acetylcholine receptor M1; MC_4_R, melacortin 4 receptor; mGluR_5_, metabotropic glutamate receptor 5; S1P, sphingosine‐1‐phosphate receptors; Smo, smoothened receptor; TMZ, temozolomide; THC, tetrahydrocannabinol.

**TABLE 2:** Potential GPCRs therapies for AD and GMB.

Therapeutic target	Drug candidate	Type of ligand	Therapy	References
**Alzheimer's Disease**				
M_1_ AChR	HTL9936	Partial agonist	HTL9936 demonstrated favourable effects on memory centres in humans at doses that effectively mitigated the adverse effects that hindered previous attempts to target this receptor in AD.	[51]
H_3_R	GSK239512	Antagonist	GSK239512 had modest and selective effects on the cognitive function of patients with mild‐to‐moderate AD.	[147]
CXCR2	SB225002	Antagonist	SB225002 inhibits CXCR2‐mediated γ‐secretase activity, which not only contributes to reducing Aβ levels but also has the potential to alleviate CXCR2‐mediated inflammation that occurs in AD and worsens its progression.	[138]
D_2_R	Bromocriptine	Agonist	Bromocriptine activates D_2_R, promoting PP2A and JNK recruitment by β‐arrestin 2. This inhibited JNK‐mediated transcription of proinflammatory cytokines and NLRP3 inflammasome activation in microglia. Thus, Bromocriptine alleviates Aβ1‐42‐induced neuroinflammation and memory deficits via the D_2_R/β‐arrestin 2/PP2A/JNK signalling axis.	[133]
5‐HT_6_	EMD‐386088	Agonist	EMD‐386088, which targets the 5‐HT_6_ receptor, demonstrated neuroprotection against Aβ25‐35‐induced toxicity. This effect was achieved by reducing ROS levels and preventing impairment of neurite outgrowth.	[136]
5‐HT_6_	SB‐399885	Antagonist	SB‐399885, which targets the 5‐HT_6_ receptor, has demonstrated neuroprotection against Aβ_25‐35_‐induced toxicity. This effect was achieved by reducing ROS levels and preventing impairment of neurite outgrowth.	[136]
M_1_ AChR	VU0364572	Agonist	VU0364572 significantly reduced cortical levels of oAβ, indicating a potential mechanism by which VU0364572 exerts its neuroprotective effects	[131]
M_1_ AChR	VU0486846	Positive Allosteric	Activation of M_1_ mAChR with VU0486846 reduces Aβ plaques, microgliosis, and astrocyte reactivity around hippocampal plaques in female APP/PS1 mice.	[129], [130]
**Glioblastoma Multiforme (GBM)**				
MC_4_R	ML00253764	Antagonist	Concurrent administration of TMZ and ML00253764 resulted in a marked synergistic effect on glioblastoma cells. In an in vivo setting, this combination demonstrated a robust and noteworthy reduction in GBM tumour volumes.	[148]
NK1R	Aprepitant	Antagonist	Aprepitant can enhance the cytotoxicity of TMZ and Ritonavir, suggesting that it can be added to the Stupp protocol.	[149]
CXCR7	CCX771	Antagonist	CCX771 has been shown to be a potential route to decrease the spread of tumour cells, their metastasis, and angiogenesis.	[144]
CXCR4	AMD3100‐SPNPs	Antagonist	AMD3100‐SPNPs effectively inhibited CXCL12/CXCR4 signalling in vitro in mouse and human GBM cell cultures. This inhibition was also observed in an in vivo mouse model of GBM. Furthermore, the combination of AMD3100‐SPNPs and radiation treatment resulted in prolonged survival.	[142]
CB_1_	CP55‐940	Agonist	CP 55–940 showed cell death in glioblastoma cell lines via apoptotic mechanisms.	[150]
CB_1_	WIN 55212‐2	Agonist	WIN 55,212–2 only moderately decreased the viability of glioblastoma cells.	[150]
CB_1_	SR141716	Antagonist	The upregulation of MICA/B induced by SR141716 was directly associated with the level of CB_1_ expression and was observed exclusively in malignant glioma cells, whereas normal human astrocytes did not exhibit this effect.	[151]
CB_2_	COR167	Agonist	COR167 inhibited cell growth in vitro via an apoptosis‐independent mechanism.	[145]
S1P receptor	Fingolimod	Agonist	Fingolimod acts as an agonist of S1P receptors, inhibiting the CXCR4 receptor and its ligand, S1P.	[140]
LPA receptor	Ki6425	Antagonist	The GBM cell line inhibited hypoxia‐induced EGFR phosphorylation upon treatment with the LPA receptor inhibitor, Ki6425.	[146]
GPR17	GA‐T0	Agonist	GA‐T0 was shown to cross the BBB and reduce tumour volume due to the significant GBM cell death and apoptosis caused by GA‐T0.	[152]
GPR15	THC	Agonist	The antitumour action of THC modulated the population of Ki67+ cells in patient‐derived GBM cells. This effect was primarily mediated by the activation of orphan receptor GPR55, without significant involvement of CB_1_ and CB_2_ receptors, which are typically considered the usual targets.	[153]
GnRH	Goserelin Acetate	Agonist	GBM LN229 cells treated with the GnRH agonist Goserelin acetate showed a reduction in cell proliferation up to 48.2%. Overexpression of KNG1 and significant inhibition of EGFR were observed after treatment, leading to the hypothesis of a possible link between GnRH signalling and EGFR signalling pathways via KNG1.	[154]
GPR68	Ogremorphin (OGM)	Antagonist	GPR68 inhibition by OGM induces ferroptosis in GBM cells through the upregulation of ATF4 and its downstream target CHAC1.	[155]

Abbreviations: 5‐HT_6_, 5‐HT Receptor 6; AD, Alzheimer's Disease; BBB, Blood–Brain Barrier; CB_1_, cannabinoid receptor Type 1; CB_2_, cannabinoid receptor Type 2; CXCR2, chemokine receptor Type 2; CXCR4, CHEMOKINE RECEPTOR Type 4; CXCR7, chemokine receptor Type 7; D_2_R, dopamine receptor D2; GBM, Glioblastoma Multiforme; GnRH, gonadotropin‐releasing hormone receptor; GPR15, G‐protein coupled receptor 15; GPR17, uracil nucleotide/cysteinyl leukotriene receptor; GPR68, ovarian cancer G‐protein coupled receptor 1; H_3_R, histamine receptor H3; LPA, lysophosphatidic acid; M_1_ AChR, muscarinic acetylcholine receptor M1; MC_4_R, melacortin 4 receptor; NK1R, neurokinin‐1 receptor; oAβ, oligomeric amyloid‐Beta; S1P, sphingosine‐1‐phosphate; THC, tetrahydrocannabinol; TMZ, temozolomide.

## 7 Advancing GPCR‐Targeted Therapies: Challenges, Innovations and Future Directions

GPCRs act as highly plastic signalling hubs, whose biological effects are strongly shaped by cellular context, disease state and age. This context dependence is particularly critical when considering GPCR‐targeted interventions for AD and GBM, where identical receptors and downstream pathways can either promote neuronal survival or drive malignant growth. Harnessing this duality requires therapeutic strategies that go beyond simple receptor activation or inhibition and exploit the signalling bias, cell type specificity and disease stage dependence.

A major challenge shared by both disorders is the effective drug delivery to the CNS. The BBB restricts the diffusion of most small molecules and biologics, and age‐associated changes in vascular integrity, neuroinflammation, and transporter expression further complicate pharmacokinetics [113]. In GBM, these constraints are compounded by intratumoural heterogeneity, genomic instability, and adaptive resistance mechanisms that rapidly rewire signalling networks in response to therapy [5, 39]. In AD, diffuse pathology, synaptic vulnerability, and network‐level dysfunction impose similar barriers, albeit through fundamentally different biological processes. These constraints underscore the need for therapies that are not only target‐specific but also resilient to the dynamic environment imposed by ageing and disease progression.

The intrinsic complexity of GPCRs, which adopt multiple active conformations and engage diverse signalling partners, enables biased signalling that selectively activates protective or deleterious pathways, aggravating therapeutic development [7, 25, 156]. While this offers opportunities to fine‐tune therapeutic responses, it also poses the risk of unintended effects, particularly when a receptor supports opposing outcomes in neurodegeneration and cancer therapy. Here, defining what constitutes “therapeutic bias” in ageing tissues, where baseline signalling, metabolic state, and inflammatory tone are altered, remains a central question.

Recent advances in structural biology and computational modelling have begun to address these challenges. High‐resolution GPCR structures obtained by cryo‐EM and X‐ray crystallography, combined with AI‐based structure prediction and molecular dynamics simulations, now enable the rational design of conformation‐specific and pathway‐biased ligands [100, 120, 122, 123, 126, 157]. These approaches allow the study of receptor dynamics under conditions that closely approximate disease‐relevant states, supporting target prioritisation, hypothesis generation, and structure‐based optimisation of ligands that may stabilise neuroprotective signalling in AD or suppress oncogenic pathways in GBM. However, translating such precision into robust in vivo efficacy requires systematic validation across various cell types and disease stages.

Innovations in experimental modelling and drug delivery further support this translational pipeline. Nanoparticle‐based and carrier‐assisted delivery systems improve brain penetration and enable controlled release [113, 158], whereas patient‐derived and induced pluripotent stem cell‐based organoids provide physiologically relevant platforms for evaluating GPCR‐targeted interventions [159, 160]. AD organoids capture neuron–glia interactions, synaptic decline, and metabolic stress [161, 162, 163], whereas GBM organoids preserve tumour heterogeneity, invasive behaviour and hypoxic niches [164, 165, 166]. Combining these models with computational prediction creates an iterative framework in which GPCR ligands can be optimised, tested, and refined under conditions that reflect age‐associated biology of the target [167].

Ultimately, advancing GPCR‐targeted therapies for CNS disorders will require the integrated alignment of structural biology, artificial intelligence (AI), disease‐relevant model systems and ageing biology. Rather than viewing AD and GBM as mechanistically unrelated entities, recognising them as context‐dependent manifestations of GPCR network dysregulation reframes drug development around signalling plasticity, the cellular environment and disease stage. This perspective emphasises ageing as a critical modulator of GPCR function and opens the door to precision strategies capable of exploiting shared molecular frameworks while delivering disease‐specific outcomes in the ageing brain.

## 8 Conclusion

AD and GBM occupy opposite poles of cell cycle control: neurones in AD re‐enter the cell cycle only to die, whereas GBM cells proliferate without regulation. Beneath this contrast lies a shared molecular grammar of chronic neuroinflammation, metabolic imbalance and dysregulated GPCR signalling. As GPCR networks are upstream of amyloid processing, Tau phosphorylation, angiogenesis, immune evasion and metabolic rewiring, they create mechanistic and pharmacological bridges between neurodegeneration and oncogenesis that, if carefully crossed, could yield transformative therapies.

Importantly, this therapeutic bridge is inherently asymmetric and shaped by age‐dependent cellular contexts. In GBM, effective therapy requires the suppression of oncogenic signalling through antagonists or inverse agonists targeting receptors such as CXCR4, LPA₁, FPR1 and D2R. In AD, many of these receptors require biased or agonistic modulation to bolster synaptic resilience, enhance Aβ clearance, and limit chronic neuroinflammation. Mastering this context dependence, shaped by cell type, disease stage and ageing, offers a path towards precision ligands that can elicit opposite biological effects within a shared signalling framework.

This review highlights the experimental and computational tools that enable such advances, from multiomics and AI‐assisted docking to single‐cell studies and organoid validation. Yet key gaps remain. The extent and functional impact of GPCR crosstalk, mainly with receptor tyrosine kinases and ion channels, remain unclear and may crucially affect therapeutic outcomes. Moreover, the structural bases of signalling bias in diseased neural and tumour microenvironments remain ill‐defined and likely vary dynamically with ageing and disease evolution. Therefore, incorporating patient‐specific GPCR signatures into clinical trial designs may be essential for improving stratification and therapeutic response.

Progress in this field demands deeper integration of chemical biology, patient‐derived models, cryo‐EM, ML and systems neuroscience to preserve mechanistic fidelity across the in silico‐in vitro‐in vivo pipelines. Ultimately, viewing AD and GBM as context‐dependent manifestations of GPCR network dysregulation, rather than as unrelated disorders, reframes the therapeutic approach around signalling plasticity and the biological environment. This angle offers a conceptual framework for guiding the rational design of GPCR‐targeted strategies that are mechanistically informed and pathology‐specific to the ageing brain.

## Author Contributions

All authors contributed to the study conceptualization. A.B.C., S.P.A., L.F.P. and N.R.‐F. conducted the literature review and contributed to data interpretation and manuscript drafting. N.R.‐F. and I.S.M. supervised the study and contributed to critical revision, editing and final approval of the manuscript.

## Funding

This work was supported by the EU Recovery and Resilience Facility and Portuguese national funds via FCT—Fundação para a Ciência e a Tecnologia, under projects LA/P/0058/2020 [DOI: 10.54499/LA/P/0058/2020], UID/PRR/4539/2025 [DOI: 10.54499/UID/PRR/04539/2025], UID/04539/2025 and 2024.07255. IACDC [https://doi.org/10.54499/2024.07255.IACDC]. B. C. and S. A. were supported by the FCT through PhD scholarships 2022.12479.BD and 2024.03713.BDANA. The PURR.AI team also acknowledges COMPETE2030‐FEDER‐01475900 SI I&DT Individual—MPr‐2023‐09 programme under contract 18415.

## Conflicts of Interest

The authors, Irina S. Moreira and Nícia Rosario‐Ferreira, as co‐founders, and Ana B. Caniceiro and Sofia P. Agostinho, as members of the team at PURR.AI, declare that there are no patents, products in development, or marketed products associated with this study that could be construed as a conflicts of interest. Luiz F. Piochi declares no conflicts of interest.

## Data Availability

Data sharing is not applicable to this article because no datasets were generated or analysed during the current study.
